# Improvement in medical students’ knowledge on chronic pain assessment through integrative learning approaches: a randomized controlled trial

**DOI:** 10.3389/fpain.2023.1210370

**Published:** 2023-08-16

**Authors:** Ratna Farida Soenarto, Besthadi Sukmono, Ardi Findyartini, Astrid Pratidina Susilo

**Affiliations:** ^1^ Department of Anesthesiology and Intensive Care, Faculty of Medicine, Universitas Indonesia, Jakarta, Indonesia; ^2^ Department of Medical Education & Medical Education Center IMERI, Faculty of Medicine, Universitas Indonesia, Jakarta, Indonesia; ^3^ Department of Medical Education and Bioethics, Faculty of Medicine, Universitas Surabaya, Surabaya, Indonesia

**Keywords:** chronic pain assessment, PQRST, ACT-UP, pain education, randomized controlled trial

## Abstract

**Introduction:**

This study aimed to compare the knowledge and skills of medical students in chronic pain assessment after being trained using the PQRST (P, provoke and palliate; Q, quality; R, region and radiation; S, severity; T, time) and ACT-UP (A, activity; C, coping; T, think; U, upset; P, people) mnemonics with those using only the PQRST mnemonic.

**Methods:**

A double-blind, randomized controlled trial was conducted at the Faculty of Medicine, Universitas Indonesia, including forty students who participated in a simulation-based chronic pain assessment workshop. Pre- and post-test scores were used to assess participants’ knowledge. Two independent raters assessed the students’ skills.

**Results:**

No significant differences in knowledge or skills were observed between the groups; however, a significant improvement in the post-test scores (85.71 [71.43–95.24]) compared to the pre-test scores (61.90 [25.87–90.48]) was observed. The students reported high satisfaction with the workshop.

**Conclusions:**

Training with the PQRST and ACT-UP mnemonics is not better than training with the PQRST mnemonic alone in improving students’ knowledge and skills in chronic pain assessment. Nevertheless, this pain education workshop was beneficial for student learning. Learning of patient-oriented chronic pain assessment should be provided in a repetitive and integrative fashion using different approaches, such as lectures, demonstrations, simulations, and interactions with patients experiencing chronic pain. To conclude, mnemonics are helpful but not a primary learning tool.

## 1. Introduction

Chronic pain affects psychological conditions, reduces productivity and daily activity, and significantly affects a patient's social and economic status (1, 2). The prevalence of chronic pain varies worldwide, with an estimate of 10.1%–55.2% of the adult populations, indicating that pain management initiatives frequently face barriers (1, 3).

One of these barriers is the lack of knowledge and skills among health professionals to comprehensively understand the subjective pain experienced by patients (4). Studies have shown that the competence of health professionals in pain-related assessment is inadequate (5, 6), and pain education is not a priority in their training curricula (7). Therefore, the paradigm of pain learning should change radically, focusing not only on biological aspects but also on psychosocial aspects (8, 9). An essential part of pain learning is pain assessment (10). Pain assessment is a process that involves dialogue between patients and health professionals regarding the description of pain and its intensity, patient's response to pain, and the impact of pain on patients’ lives (11). Although pain assessment has been discussed in the literature, research on this topic is still limited (12).

The PQRST mnemonic (P, provoke and palliate; Q, quality; R, region and radiation; S, severity; T, time) has been used for pain assessment in clinical practice and education (11, 13). Mnemonics offer several benefits. First, mnemonics are helpful for systematically memorizing and operationalizing concepts (14). Second, mnemonics are simple and fit well into the context of communication between patients and health professionals with time constraint (15). Nevertheless, the PQRST mnemonic focuses on the biomedical aspects of pain and is less supportive in exploring the psychosocial aspects of patients (11, 13).

Some experts have recommended the use of ACT-UP (A, activity; C, coping; T, think; U, upset; P, people) in patients with chronic pain. The ACT-UP mnemonic has an additional value in helping students conduct functional and psychosocial chronic pain assessments more comprehensively (16, 17). A combination of PQRST and ACT-UP is helpful and straightforward in guiding students to perform a comprehensive pain assessment. This can help students memorize and structure their history-taking process (18). However, the use of this combination for pain education has not yet been studied.

This study aimed to investigate whether pain assessment training using the PQRST and ACT-UP mnemonics is more effective than that with the PQRST mnemonic alone in improving the knowledge and skills of medical students. We hypothesized that pain assessment training using the PQRST and ACT-UP mnemonics is more effective in improving the knowledge and skills of medical students than the PQRST mnemonic alone. The results of this study could guide the development of pain education programs for students.

## 2. Materials and methods

We conducted a double-blind, randomized controlled trial. The study population comprised pre-clinical medical students in the Faculty of Medicine, Universitas Indonesia. Participants were chosen randomly from a list of third-year pre-clinical students. Eligibility criteria included students who completed modules on pain physiology, had basic knowledge of diseases causing pain in primary care, physical examinations, and communication skills. Students with experience in extracurricular chronic pain assessment training and those with chronic pain were excluded. Non-attendance or students who did not finish the workshops were considered as dropouts. Sample size was calculated based on a difference of five points, power of 90%, alpha of 5%, one-way, and dropout of 25%. The sample size was 40 for two groups of participants.

**Figure 1:**
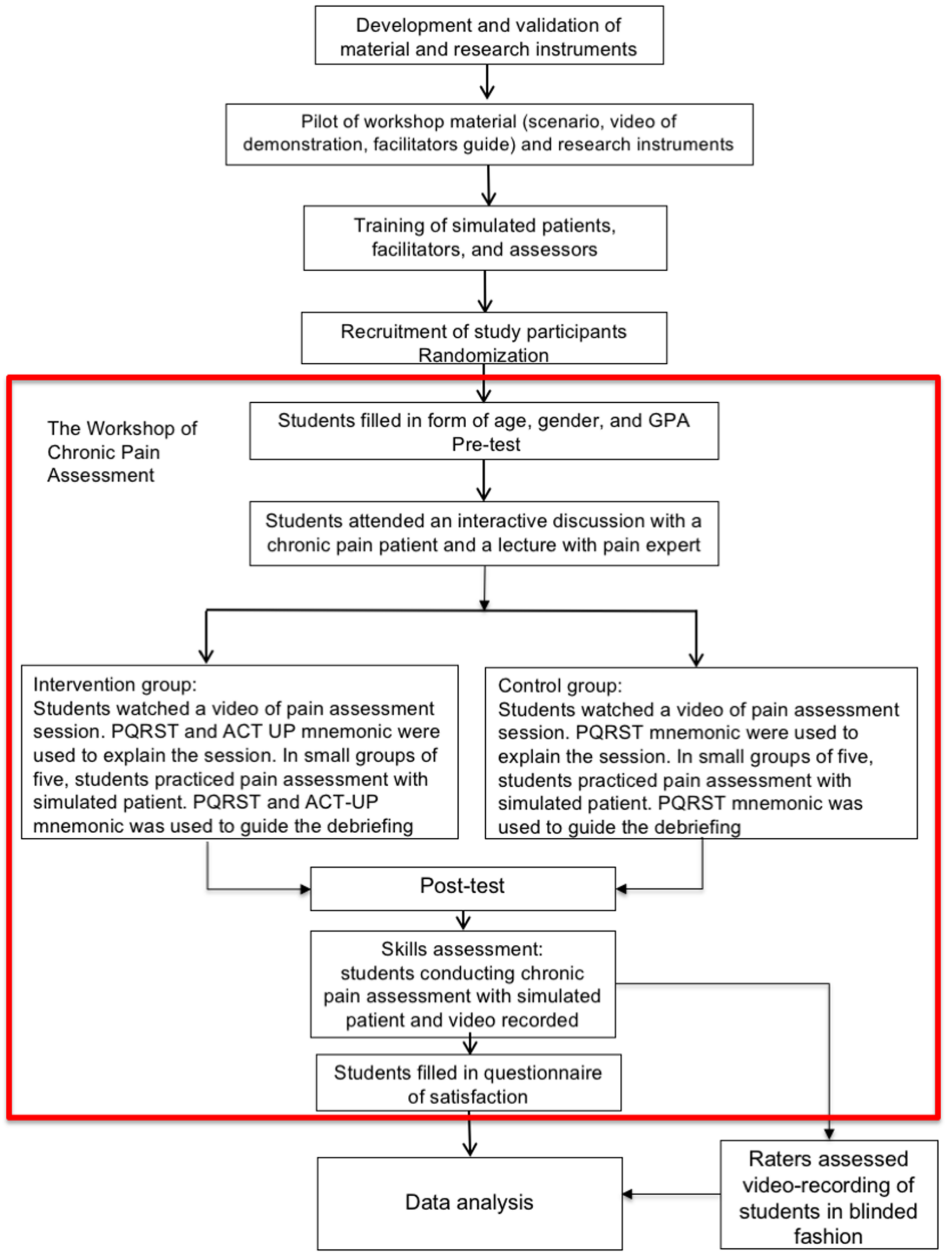
Study process.

In this study, the competence of pain assessment was in concordance with the pain curriculum of International Association for the Study of Pain (IASP) (10) and the Indonesian Standard of Competence of Medical Doctors (19). The study process is described below and summarized in Figure 1.

### 2.1. Preparation

Learning materials and research instruments were developed based on the literature and discussion among the research team. The research instruments included (i) pre- and post-test scores to assess knowledge; (ii) a checklist to assess skills; and (iii) a questionnaire to assess student's satisfaction with the training (see Supplementary Files). The skill assessment scenario involved a case of low back pain. The instruments were validated by eight experts from Indonesia, the Netherlands, and USA. They were anesthesiologist and pain management physicians, family medicine physicians, and experts in medical education and communication skills training. One of them was the inventor of the ACT-UP mnemonics. The final drafts were translated into Indonesian language and back-translated into English language by an independent translator. Cultural and language comprehension was ensured by an independent bilingual third party with a background in anesthesiology.

Three national experts conducted a content validation. Aiken's V Coefficients (range, 0–1) were calculated for each item, with a score of >0.5 considered adequate (20, 21). The Aiken's V coefficient for the knowledge test was 0.78–1; for the checklist was 0.78–1, and for the questionnaire was 0.89–1. Finally, the instruments were piloted and their reliability was measured.

We conducted preparation courses for facilitators, raters, and simulated patients through lectures, demonstrations, and practice sessions. The raters piloted the checklist and measured its reliability.

### 2.2. Intervention

We conducted a one-day integrative workshop on chronic pain assessment in the Simulation-Based Medical Education and Research Center, Indonesia Medical Education and Research Institute. An independent party conducted the randomization. The students were blinded to the group allocation, but they knew that there were two learning approaches.

To ensure that both groups received equal intervention, all students participated in the first integrative sessions together. A patient with chronic pain shared her experiences of living with pain and its influence on functional and psychosocial conditions. An expert in pain management provided interactive lectures. The ACT-UP mnemonic was not used to ensure blinding.

Thereafter, the intervention and control groups were separated into two locations to maintain blinding. Each group underwent a demonstration of chronic pain assessment using a doctor-patient simulation video. There was a significant difference between the videos of the two groups. In addition to the explanation of the PQRST mnemonics and principles of comprehensive chronic pain assessment, the intervention group received an explanation of the PQRST and ACT-UP mnemonics in the video.

Subsequently, the students practiced pain assessment in small groups of five with one facilitator. Each student practiced a one-time simulation and provided feedback to the other group members. There were four scenarios, based on diseases (chronic low back pain or headache) and functional and psychosocial problems. We provided a flipchart with information on the mnemonics*;* the intervention group obtained information about the PQRST and ACT-UP mnemonics, whereas the control group obtained information about the PQRST mnemonic only. Differences in the use of mnemonics were also noted in the feedback session.

### 2.3. Data collection

The students completed the pre- and post-tests at the beginning of the training and at the end of the workshop. They conducted a chronic pain assessment on a simulated patient with back pain, which was video-recorded. Four raters, blinded to the group allocation, assessed the video recordings of the simulations. Each student was independently assessed by a pair of raters. At the end of the training, students completed questionnaires on satisfaction.

### 2.4. Data analysis

We used Statistical Package for the Social Sciences (SPSS) version 20.0 for data analysis. An independent *t*-test was used to compare means between the groups, or the Mann–Whitney *U*-test was applied when the data were not normally distributed. We compared pre- and post-test data using the Wilcoxon signed-rank test.

### 2.5. Ethical consideration

Ethical approval was granted by the Ethical Committee of the Faculty of Medicine, Universitas Indonesia and Cipto Mangunkusumo General Hospital (0467/UN2. F1/ETIK/2018). All the participants had the right to obtain information about the study and refuse to participate. Refusal did not influence the students’ academic assessments. Students who agreed to participate signed an informed consent form.

To ensure blinding, the students were informed about the different intervention approaches in the two groups; however, they were no given detailed information about the differences. Furthermore, the information sheet and consent form did not mention the PQRST or ACT-UP mnemonics. This concealment did not pose an additional risk to the students and was approved by the Ethical Committee.

## 3. Results

**Figure 2:**
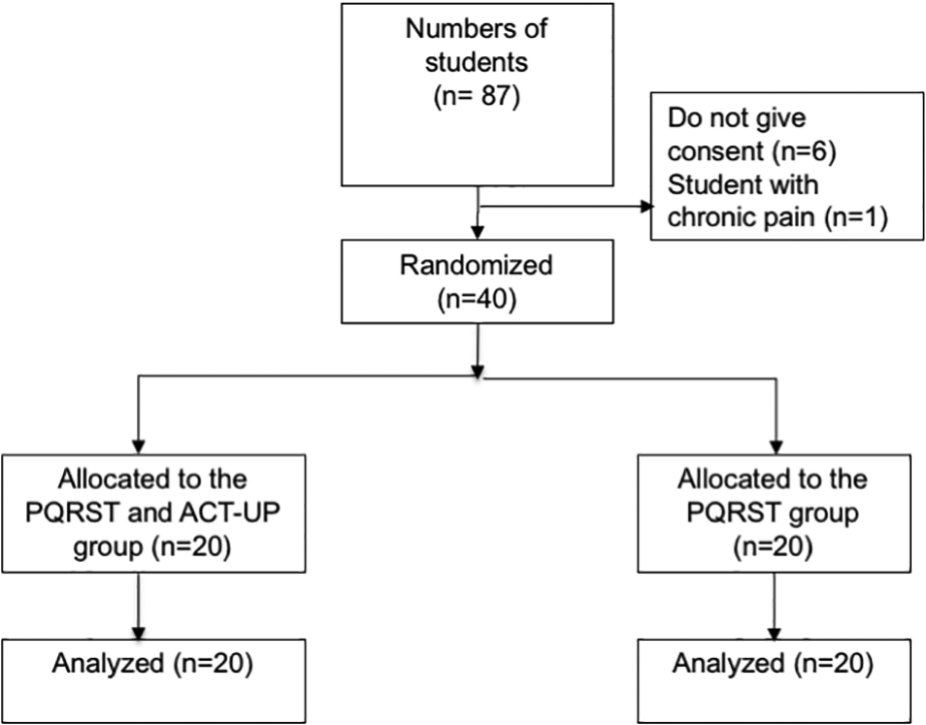
Participants’ flow chart.

The participants’ flow chart is presented in Figure 2. Table 1 shows the comparable characteristics of the participants in each group. The pre- and post-tests consisted of 21 items with a split-half reliability of 0.70, showing moderate reliability (22). Table 2 shows a comparison of students’ knowledge and skills between the two groups. Knowledge was assessed by calculating the percentage of correct responses. Difference was obtained by subtracting the pre-test score from the post-test score. This difference was *p* = 0.066 or >0.025 (one-tail hypothesis). The skill assessment was used to obtain the skill score by calculating the total score × weight × 100 divided by the maximum score. The reliability test between raters showed an adequate agreement of Intraclass Correlation (ICC) 0.76 (23). This finding indicates that the knowledge and skill levels of students trained with the PQRST and ACT-UP mnemonics were not higher than those trained with the PQRST mnemonics only.

**Table 1:** Characteristics of students in both the groups.

Characteristics	PQRST and ACT UP	PQRST
	(*n* = 20)	(*n* = 20)
Age (year)^a^	20 (SD, 1)	21 (SD, 0.5)
Gender^b^		
Male	12 (60)	12 (60)
Female	8 (40)	8 (40)
Grade point average^a^	3.39 (SD, 0.14)	3.39 (SD, 0.21)

^a^ Data are presented as mean (SD).

^b^ Data is presented in *n* (%).

**Table 2:** Comparison of students’ knowledge and skills between the two groups.

	PQRST and ACT-UP	PQRST	95% CI from the mean difference	*p*-Value
Knowledge				
Pre-test	63.81 (SD, 14.69)	66.67 (SD, 8.18)		0.183^a^
Post-test	85.95 (SD, 6.08)	82.14 (SD, 8.30)	3.17 (−1.54–7.89)	0.106^b^
Difference	22.14 (SD, 12.29)	15.47 (SD, 9.88)	5.00 (−4.71–13.80)	0.066^b^
Skills	71.92 (SD, 7.26)	74.00 (SD, 9.63)	−2.08	0.445^b^
			−7.54 (SD, 3.38)	

For the readers’ accessibility, all data are presented in mean (SD).

^a^ Mann–Whitney test.

^b^ Independent *t*-test.

However, there was a significant difference between the pooled group of 40 participants in their knowledge before and after the workshop (Table 3).

**Table 3:** Comparison of students’ pre- and post-test scores.

	Knowledge	*p*-Value
Pre-test	61.90 (25.87–90.48)	0.000^a^
Post-test	85.71 (71.43–95.24)	

^a^ Wilcoxon signed-rank test.

In the satisfaction questionnaires, the participants responded on a scale of 1–4 to the question, “How do the following items support your learning process?” (1 = not very supportive, 2 = not supportive, 3 = supportive, 4 = very supportive). The questionnaire for the intervention group consisted of 15 items, while that of the controlled group consisted of only 14 items; the item “the use of ACT-UP mnemonic” was not asked. Therefore, the alpha coefficient of reliability was calculated using the questionnaire with 14 items. The alpha coefficient was 0.76 and was considered acceptable (24). The level of satisfaction in both the groups was high, with a median score of 3.8 (3.33–4) for the PQRST and ACT-UP group and 3.75 (3.07–4) for the PQRST group. The results of the questionnaire are presented in the Supplementary Material.

## 4. Discussion

This study aimed to investigate whether the incorporation of the ACT-UP mnemonic in pain assessment training could improve the knowledge and skills of medical students. The intervention and control groups were comparable. There were no significant differences in knowledge at the beginning of the intervention. After the training, there was no difference in skills or knowledge between the intervention and control groups. This showed that training with the PQRST and ACT-UP mnemonic was not better than training with only the PQRST mnemonic.

Theoretically, mnemonics work as a tool to help memorize and structure lines of thinking (14, 18, 24). Our result differs from those of the other studies that have compared the two mnemonics during training in an emergency context, showing that mnemonics are superior in supporting memory and organizing the causes of emergencies (14). The ACT-UP mnemonic, consisting of functional and psychosocial items, potentially helps students perform a comprehensive chronic pain assessment. However, our study showed that even without the ACT-UP mnemonic, students in the control group could perform a comprehensive pain assessment. Our results also showed a significant difference between the pre- and post-test scores and high post-test and skills test scores of the pooled group of 40 students.

These findings indicate that in our study, an additional mnemonic may not be necessary to improve students’ learning, or that our measurements may not have the sensitivity to illuminate the psychosocial and functional strengths of the ACT-UP mnemonic. We believe that the integrative approaches, structured from simple to complex, consisting of various methods, including talk shows with real patients, expert lectures, demonstrations, and simulations, are beneficial and adequate as learning tools. The students’ improvement was also attributed to the reinforcement of chronic pain assessment principles across various learning activities (25). A systematic review has shown that simulations can improve students’ skills (26), while interactions with patients with chronic pain provide exposure to real-world scenarios (27, 28). This result was consistent with the high satisfaction of students in both the groups. Students reported that the different integrative approaches used in this workshop supported learning.

This is the first empirical study on the ACT-UP mnemonic. Previous studies on the ACT-UP mnemonic have not included empirical data (16, 17). Additionally, previous studies on pain learning did not use control groups (29) or blinding (12, 28). Thus, the internal validity of this study was adequate. An independent party conducted the group allocation, and blinding was maintained for both the groups (23). Expert validation showed that the items measuring knowledge, skills, and satisfaction had good content validation. The reliability of these instruments is moderate and reasonable (24).

This study has some limitations. First, we limited the training to one day in order to maintain blinding and prevent students from communicating the differences between the interventions. Additionally, repeating the simulation was also difficult, owing to time constraints. Each student was able to conduct the simulation once and participate in the other four simulations in a group. Therefore, we could not assess the skills retention (30). Long-term training evaluation can be conducted when students are exposed to real patients during their clinical rotations. Second, this study was conducted at a single institution, and the adoption of this study should take into consideration the curriculum and student characteristics. Future studies should be conducted in other institutions, involving other health professionals or in a continuing education context.

In conclusion, to improve the knowledge and skills of medical students, training with the PQRST and ACT-UP mnemonics is not superior to that with the PQSRT mnemonic alone. Mnemonics are helpful, but they are not a primary learning tool. Patient-oriented chronic pain assessment learning should be integrated and provided repetitively using different approaches, such as lectures, demonstrations, simulations, and interaction with patients experiencing chronic pain.

## Data Availability

The raw data supporting the conclusions of this article will be made available by the authors, without undue reservation.
