# Supplementary material for: Improvement in medical students’ knowledge on chronic pain assessment through integrative learning approaches: a randomized controlled trial
Source: Front Pain Res (Lausanne). 2023 Aug 16;4:1210370. doi: 10.3389/fpain.2023.1210370 (PMC10469308; doi:10.3389/fpain.2023.1210370)
Supplement: Supplementary file 1 [file Datasheet1.docx]

**Supplementary Files**

***PQRST Mnemonic***

| P = *Provoke* and *Palliate*  What are the causes of pain? What provokes and palliates the pain? Have you obtained any medication, have you taken them regularly, and do they help palliate the pain?  Q = *Quality*  How is the description of the pain? Is it sharp or dull? Do you feel a burning sensation?  R = *Region* and *Radiation*  Where do you feel the pain? Is it radiating and where? Do you feel the pain in other locations?  *S = Severity*  How is the intensity of the pain? How does pain influence your sleep, physical function, work, mood, family life, social, or sexual life?  T = *Time* (or *T*emporal)  When do you feel the pain? In the evening or afternoon? Is it intermittent or continuous? How long does the pain occur? When does it relieve? |
| --- |

Modified from Powell RA et al. [11]

***ACT-UP Mnemonic***

| A = *Activities*  How pain influence daily patient activities such as sleeping, eating, or social life?  C = *Coping*  How does the patient cope? What makes the pain increase or reduce?  T = *Think*  What does the patient think about the pain? Would it be better?  U = *Upset*  How does the patient feel about the pain? Does the pain make the patient sad or depressed?  P = *People*  How people around patients response when the patient is in pain? |
| --- |

Modified from Dansie E and Turk D [16]

**Students’ Satisfaction of the Workshop**

| Item | PQRST and ACT-UP | PQRST |
| --- | --- | --- |
| ACTIVITY |  |  |
| Discussion with chronic pain patient | 3 (2-4) | 3.5 (2-4) |
| Expert lecture | 4 (3-4) | 4 (3-4) |
| Demonstration of chronic pain assessment | 4 (3-4) | 4 (3-4) |
| Simulation of chronic pain assessment | 4 (3-4) | 4 (3-4) |
| COMPONENT |  |  |
| Use of PQRST *mnemomic* | 4 (1-4) | 4 (3-4) |
| Use of ACT-UP *mnemonic* | 4 (1-4) | N/A |
| Role of patient simulation | 4 (2-4) | 4 (2-4) |
| Role of facilitator in simulation | 4 (3-4) | 4 (3-4) |
| Variation of scenarion in simulation | 3 (2-4) | 3 (1-4) |
| *Debriefing* after simulation (feedback session) | 4 (3-4) | 4 (3-4) |
| ORGANIZATION |  |  |
| Venue | 4 (3-4) | 4 (2-4) |
| Facility (audiovisual media, layout of simulation room) | 4 (3-4) | 4 (2-4) |
| Time management | 4 (3-4) | 4 (2-4) |
| Interaction with other workshop participant | 4 (3-4) | 4 (2-4) |
| RELEVANCE |  |  |
| The relevance of this training to the future need of learning | 4 (3-4) | 4 (4-4) |
| Median Score | 3.8 (3.33-4) | 3.75 (3.07-4) |
| *data are presented in median (minimum-maximum) |  |  |

**Research Instruments**

Knowledge Test

| 1. | What is pain?   1. pain is a genetic process that has relations with the patient's emotional processes that interfere with the activities 2. pain is a sensory and motoric experience that is not related to the patient’s psychosocial condition 3. pain is a symptom of pathognomonic of ongoing and progressive tissue damage 4. **pain is a sensory and emotional experience that may be followed by visible tissue damage** |
| --- | --- |
| 2. | What is ‘transduction’ in the basic mechanism of pain?   1. The process of impulse inhibition of pain in the spinal cord 2. The process of release of pain mediators in the cerebral cortex 3. The process of impulse delivery by peripheral nerve fibers 4. **The process of changing the pain stimulus into electrical energy** |
| 3. | What is ‘transmission’ in the basic mechanism of pain?   1. The process of pain impulse facilitation in the brain 2. **The process of delivering pain impulses by the nerve fibers** 3. The process of pain mediator release in the spinal cord 4. The process of changing the pain stimulus into electrical energy in the cerebral cortex |
| 4. | What is ‘allodynia’?   1. Transmission of pain in the surrounding nerve cells that does not get pain stimulus 2. **Perception of pain in the stimulus that normally does not cause pain** 3. The process of changes in the central nervous system due to noxious stimuli   d. The increasing pain response towards the stimulus beyond the normal response |
| 5. | What is neuropathic pain like?   1. Blunt sensation c. Sharp sensation 2. Gripped sensation d. Burning sensation |
| 6. | What are the characteristics of visceral pain?   1. **Visceral pain can be accompanied by autonomic reflects such as nausea and vomiting** 2. Visceral pain is often felt as sharp pain or electrical shock 3. Visceral pain is associated with skin or bone damage 4. Viseral pain is localized and does not spread |

**7.** In PQRST mnemonic, what question is important to ask in relation to “R”?

- 1. **The treatments that have been received and the impacts on the pain**
  2. The scale of pain assessed using the Numeric Rating Scale
  3. The spread and the location of pain felt by the patient
  4. The characteristics of pain suffered by the patient

**8.** What kind of scale of pain is this?


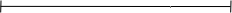


*No pain Worst*

*imaginable pain*

1. Wong Baker Rating Scale
2. Numerical Rating Scale
3. **Visual Analogue Score**
4. Faces Pain Scale

9. What nervous fibers are responsible for sharp pain?

a. A-alpha

b. A-beta

**c. A-delta**

d. A-gamma

10. When dealing with patients with chronic pain, what is being questioned with the question "Do you think your pain will get better?"

1. Patient’s ability to cope with the pain
2. **Patient’s expectation on the pain management**
3. Patient’s preference on pain management
4. Patient’s different approach in coping with the pain

11. The assessment of chronic pain is based on several principles. There are four principles listed below. Which principle is most appropriate for the assessment of comprehensive chronic pain?

1. In order to get the correct assessment, anamnesis should emphasize on the aspects of pain biomedicine.
2. **Patient’s response on the pain is as important as the impact of the pain on the patient’s life.**
3. The description of pain is more important than the severity of the pain.
4. The discussion of pain management should involve family members.

**The following vignette is the information to answer question number 12-16**

You are a physician in a community health center. Ms. Rina, a traditional herb seller, 53 years old, is complaining about having a back pain. This woman is feeling pain with the VAS 5 (scale 1-10) when sitting too long. The pain felt was like burning, spreading towards the right leg. Ms. Rina is complaining for having the pain for the last 4 months, but she just got a chance to visit the doctor today. She has been using traditional rub oil on her back to reduce the pain.

12. What kind of pain is Ms. Rina having?

1. **Neuropathic pain**  b. Somatic pain

c. Visceral pain d. Acute pain

13. You are conducting a physical examination to Ms. Rina. After doing the inspection and palpation, you are conducting the following test. What kind of test is that?

1. Distraction test
2. Faber Test

**c. Laseque test**

d. Freiberg’s maneuver

14. If Bu Rina’s test comes back positive, what things should be considered?

1. There are inferior paraparesis derived from the spinal cord disease
2. **There is an involvement of sciatic nerve on this pathological process**
3. There are neuropathic components on the patient’s pain
4. There is an inflammation in the facet joints

15. Ms. Rina is complaining of pain with VAS 5 (of 10) if she is carrying her merchandise too long.

Below is a part of the conversation between Ms. Rina and her doctor.

*R1: Doc, I cannot work if I feel painful. How can I pay for my child’s tuition?*

*D1: I understand… What do you do, mam?*

*R2: I sell traditional herb. I walk all day carrying the herb on my back. I usually work from morning until night. Because of the pain, I can only stand up until noon.*

*D2: Oh… It must be hard on you …*

*R3: Yes, I am worried about this pain. If it gets worse and I cannot walk, I will not be able to work anymore.*

In this conversation, why is it important to ask “What do you do, mam?” (D1)

a. To find out Ms. Rina’s emotion related to her child’s tuition

b. To find out Ms. Rina’s expectation related to the pain management choices

c. To find out Ms. Rina’s ability to pay for the medical costs related to the pain management

**d. To find out the impact of the pain on Ms. Rina’s functional status**

16. Ms. Rina says, “If it gets worse and I cannot walk, I will not be able to work anymore” (R3). What is reflected from the statement?

1. Patient’s activity related to the pain
2. Patient’s emotion related to the pain
3. **Patient’s perception related to the pain**
4. Coping mechanism related to the pain

**The following vignette is the information to answer questions number 18-21**

Iwan, a 20-year-old university student, is complaining about a headache. The pain has come and gone for 5 months, but it was often ignored. When the pain comes, he feels nausea and sometimes vomits. The headache is on the right side of the head, accompanied by nausea and vomiting. The frequency is 1-2x/week, the pain is VAS 5 (scale 1-10). Iwan feels better when resting and being a quiet and dimly lit place.

17. In PQRST mnemonic, what question is important to ask in relation to “R”?

a. When does the pain intensify? Morning? Afternoon? Evening?

b. What is the characteristic of the pain?

c. **How is the spread of the pain?**

**d.** How intense is the pain?

18. In PQRST mnemonic, what question is important to ask in relation to “Q”?

a. Does the patient have a history of hypertension and diabetes?

b. Does the patient feel better after taking paracetamol?

**c. Is there a burning sensation or an electric shock sensation?**

d. Is there a history of pain in the patient’s family?

19. On the physical examination, you perform a cranial nerve examination. How is the nerve VII examination done?

1. Asking the patient to glance to the right and to the left
2. Check the patient’s hearing ability
3. **Ask the patient to frown**
4. Performing a prick test on the face

20. Iwan is complaining that in the last two months, he has not been able to study well because he has a headache. As his doctor, you would like to find out further information. What are you going to ask first related to this complain?

1. Find out more about who can support Iwan to help with his study
2. Find out more about the response of the family on his study development
3. Find out more about what kind of pain Iwan feels
4. **Find out more about how the pain disturbs his daily activity**

*21.* Iwan told his doctor, “Doc, my headache got worse when I heard my friends playing guitar, **so I asked them to play guitar when I was not around**. However, I missed the time to play with my friends, and I am afraid of losing them.” What does this statement reflect?

1. The effect of pain on patient’s emotion
2. **Patient’s coping mechanism**
3. Patient’s thoughts related to the pain
4. The response of the surrounding people on the patient’s pain experience

| **SCORING**  **Checklist of chronic pain assessment skill**  **ITEM** | **0** | **1** | **2** | **3** | **WEIGHT** |
| --- | --- | --- | --- | --- | --- |
| 1. Anamnesis description and pain intensity | Student did not perform any action | Student performed 1-2 items below correctly:   1. Finding out the factors that increase or reduce the pain *(Provoke and Palliate)* 2. Finding out the description of pain *(Quality)* 3. Finding out the location and spread of the pain *(Region and Radiation)* 4. Finding out the severity of the pain, including assessing the scale of the pain *(Severity)* 5. Finding out the onset, duration and time-specific occurrence of the pain (*Time)* | Student performed 3-4 items below correctly:   1. Finding out the factors that increase or reduce the pain *(Provoke and Palliate)* 2. Finding out the description of pain *(Quality)* 3. Finding out the location and spread of the pain *(Region and Radiation)* 4. Finding out the severity of the pain, including assessing the scale of the pain *(Severity)* 5. Finding out the onset, duration and time-specific occurrence of the pain (*Time)* | Student performed all items below correctly:   1. Finding out the factors that increase or reduce the pain *(Provoke and Palliate)* 2. Finding out the description of pain *(Quality)* 3. Finding out the location and spread of the pain *(Region and Radiation)* 4. Finding out the severity of the pain, including assessing the scale of the pain *(Severity)* 5. Finding out the onset, duration and time-specific occurrence of the pain (*Time)* | 2 |
| 1. Functional and Psychosocial anamnesis condition | Student did not perform any action | Student performed 1-2 items below correctly:   1. Finding out the effect of pain towards patient’s activity *(Activity)* 2. Finding out how the patient cope with the pain *(Coping)* 3. Finding out what the patient’s wish about the pain *(Think)* 4. Finding out the patient’s emotion in dealing with the pain *(Upset)* 5. Finding out the attitudes of the family and the people around the patient when he/she is in pain *(People)* | Student performed 3-4 items below correctly:   1. Finding out the effect of pain towards patient’s activity *(Activity)* 2. Finding out how the patient cope with the pain *(Coping)* 3. Finding out what the patient’s wish about the pain *(Think)* 4. Finding out the patient’s emotion in dealing with the pain *(Upset)* 5. Finding out the attitudes of the family and the people around the patient when he/she is in pain *(People)* | Student performed all items below correctly:   1. Finding out the effect of pain towards patient’s activity *(Activity)* 2. Finding out how the patient cope with the pain *(Coping)* 3. Finding out what the patient’s wish about the pain *(Think)* 4. Finding out the patient’s emotion in dealing with the pain *(Upset)* 5. Finding out the attitudes of the family and the people around the patient when he/she is in pain *(People)* | 2 |
| 1. Physical Examination | Student did not mention physical examination | Student mentioned 1-2 physical examinations below:   1. Gait inspection and back area 2. Palpation of painful back area, Range of Motion and Test Laseque 3. Patellar and Achilles reflexes | Student mentioned 3-4 physical examinations below:   1. Gait inspection and back area 2. Palpation of painful back area, Range of Motion and Test Laseque 3. Patellar and Achilles reflexes | Student mentioned all physical examinations below:   1. Gait inspection and back area 2. Palpation of painful back area, Range of Motion and Test Laseque 3. Patellar and Achilles reflexes | 1 |
| 1. Problem analysis | Student did not mention the problem of the patient | Student mentioned one patient’s problem correctly:   1. Chronic lower back pain (student may add comparative diagnosis) 2. Functional interference (for example: interfere with the work as a driver) 3. Psychosocial interference (for example: feeling to be a burden for friends, fear of reduced income or the possibility of being paralyzed and sexual problem) | Student mentioned two patient’s problems correctly:   1. Chronic lower back pain (student may add comparative diagnosis) 2. Functional interference (for example: interfere with the work as a driver) 3. Psychosocial interference (for example: feeling to be a burden for friends, fear of reduced income or the possibility of being paralyzed and sexual problem) | Student mentioned all patient’s problems correctly:   1. Chronic lower back pain (student may add comparative diagnosis) 2. Functional interference (for example: interfere with the work as a driver) 3. Psychosocial interference (for example: feeling to be a burden for friends, fear of reduced income or the possibility of being paralyzed and sexual problem) | 2 |
| 5.Nonfarmako-logical management | Student did not give management | 1 correct item:   1. Answering the problem (for example: stay active and do exercise) 2. Able to perform 3. Easy to understand | 2 correct items:   1. Answering the problem (for example: stay active and do exercise) 2. Able to perform 3. Easy to understand | All correct items:   1. Answering the problem (for example: stay active and do exercise) 2. Able to perform 3. Easy to understand | 1 |
| 6. Communication | Student did not show a good communication skill | Student showed communication skill by applying one principle below:   1. Developing empathy with the patient using verbal communication 2. Developing empathy with the patient using verbal communication 3. Actively listening in order to find a holistic and comprehensive health problem 4. Involving the patient in making management decision | Student showed communication skill by applying two or three principles below:   1. Developing empathy with the patient using verbal communication 2. Developing empathy with the patient using verbal communication 3. Actively listening in order to find a holistic and comprehensive health problem 4. Involving the patient in making management decision | Student showed communication skill by applying all principles below:   1. Developing empathy with the patient using verbal communication 2. Developing empathy with the patient using verbal communication 3. Actively listening in order to find a holistic and comprehensive health problem 4. Involving the patient in making management decision | 1 |
| 1. Professional behavior | Student did not show any professional behavior | Student showed professional behavior as reflected in one of the following items:   1. Paying attention to self and patient’s safety (such as washing hands at the beginning of consultation) 2. Showing respect to the patient throughout the consultation session 3. Understand the limitations as general practitioner and recommend consultation to a specialist. | Student showed professional behavior as reflected in two of the following items:   1. Paying attention to self and patient’s safety (such as washing hands at the beginning of consultation) 2. Showing respect to the patient throughout the consultation session 3. Understand the limitations as general practitioner and recommend consultation to a specialist. | Student showed professional behavior as reflected in three of the following items:   1. Paying attention to self and patient’s safety (such as washing hands at the beginning of consultation) 2. Showing respect to the patient throughout the consultation session 3. Understand the limitations as general practitioner and recommend consultation to a specialist. | 1 |

**Student’s satisfaction questionnaire**

| No | How are the following items support your learning process?  Mark ‘v’ on the most appropriate answer | 1 | 2 | 3 | 4 |
| --- | --- | --- | --- | --- | --- |
|  | ACTIVITY |  |  |  |  |
| 1 | Discussion with patient with chronical pain |  |  |  |  |
| 2 | Lecture from the expert |  |  |  |  |
| 3 | Demonstration of pain assessment |  |  |  |  |
| 4. | Simulation of pain assessment |  |  |  |  |
|  | COMPONENTS |  |  |  |  |
| 5. | The use of *mnemonic* PQRST |  |  |  |  |
| 6. | The use of *mnemonic* ACT-UP |  |  |  |  |
| 7. | The role of the patient in the simulation |  |  |  |  |
| 8. | The role of the facilitator in the simulation |  |  |  |  |
| 9. | Variation of the scenario in the simulation |  |  |  |  |
| 10. | *Debriefing* after the simulation |  |  |  |  |
|  | TRAINING ORGANIZATION |  |  |  |  |
| 11. | Venue |  |  |  |  |
| 12. | Training facility (audio-visual media, simulation room layout) |  |  |  |  |
| 13. | Training time management |  |  |  |  |
| 14. | Interaction with other training participants |  |  |  |  |
|  | RELEVANCE |  |  |  |  |
| 15. | Relevance of the training with your learning needs in the future |  |  |  |  |

Scale 1 – 4 (1=does not support, 2=support, 3=support, 4=highly support)
